# Magnetic resonance spectroscopy detects differential lipid composition in mammary glands on low fat, high animal fat versus high fructose diets

**DOI:** 10.1371/journal.pone.0190929

**Published:** 2018-01-11

**Authors:** Dianning He, Devkumar Mustafi, Xiaobing Fan, Sully Fernandez, Erica Markiewicz, Marta Zamora, Jeffrey Mueller, Joseph R. Sachleben, Matthew J. Brady, Suzanne D. Conzen, Gregory S. Karczmar

**Affiliations:** 1 Department of Radiology, The University of Chicago, Chicago, Illinois, United States of America; 2 Sino-Dutch Biomedical and Information Engineering School, Northeastern University, Shenyang, China; 3 Department of Medicine, Section of Adult and Pediatric Endocrinology, Diabetes and Metabolism, The University of Chicago, Chicago, Illinois, United States of America; 4 Department of Pathology, The University of Chicago, Chicago, Illinois, United States of America; 5 Biomolecular NMR Core Facility, The University of Chicago, Chicago, Illinois, United States of America; 6 Department of Medicine, Hematology/Oncology, Hematology/Oncology, The University of Chicago, Chicago, Illinois, United States of America; University of Illinois, UNITED STATES

## Abstract

The effects of consumption of different diets on the fatty acid composition in the mammary glands of SV40 T-antigen (Tag) transgenic mice, a well-established model of human triple-negative breast cancer, were investigated with magnetic resonance spectroscopy and spectroscopic imaging. Female C3(1) SV40 Tag transgenic mice (n = 12) were divided into three groups at 4 weeks of age: low fat diet (LFD), high animal fat diet (HAFD), and high fructose diet (HFruD). MRI scans of mammary glands were acquired with a 9.4 T scanner after 8 weeks on the diet. ^1^H spectra were acquired using point resolved spectroscopy (PRESS) from two 1 mm^3^ boxes on each side of inguinal mammary gland with no cancers, lymph nodes, or lymph ducts. High spectral and spatial resolution (HiSS) images were also acquired from nine 1-mm slices. A combination of Gaussian and Lorentzian functions was used to fit the spectra. The percentages of poly-unsaturated fatty acids (PUFA), mono-unsaturated fatty acids (MUFA), and saturated fatty acids (SFA) were calculated from each fitted spectrum. Water and fat peak height images (maps) were generated from HiSS data. The results showed that HAFD mice had significantly lower PUFA than both LFD (p < 0.001) and HFruD (p < 0.01) mice. The mammary lipid quantity calculated from ^1^H spectra was much larger in HAFD mice than in LFD (p = 0.03) but similar to HFruD mice (p = 0.10). The average fat signal intensity over the mammary glands calculated from HiSS fat maps was ~60% higher in HAFD mice than in LFD (p = 0.04) mice. The mean or median of calculated parameters for the HFruD mice were between those for LFD and HAFD mice. Therefore, PRESS spectroscopy and HiSS MRI demonstrated water and fat composition changes in mammary glands due to a Western diet, which was low in potassium, high in sodium, animal fat, and simple carbohydrates. Measurements of PUFA with MRI could be used to evaluate cancer risk, improve cancer detection and diagnosis, and guide preventative therapy.

## Introduction

The increased risk for breast cancer in women is attributed to many factors including a higher body mass index (BMI) [1–3]. Although many factors influence body weight, an unhealthy diet is one of the main causes. Diets, particularly those that are high in fats and sugar, have a large impact on BMI [4]. Obesity has different impacts on the risk of aggressive breast cancer in premenopausal versus postmenopausal women [5]. This could be a contributing factor to the higher incidence rate of triple-negative breast cancers observed among younger African American and Hispanic women [6, 7]. Sieri et al. showed a weak positive association between saturated fat intake and breast cancer risk, but the association was more pronounced for postmenopausal women who had never used hormone therapy [8]. Therefore, quantitative measurements of fat content and fatty acid composition may be important for clinical assessment of cancer risk.

Proton magnetic resonance spectroscopy (^1^H-MRS) is widely used to measure lipid content and fatty acid composition [9, 10], e.g. for measurement of liver fat [11–13]. Higher magnetic field strengths (> 4.7 T) provide higher spectral resolution and this allows more detailed measurements of lipid composition [14, 15]. Recently, ^1^H-MRS has also been applied clinically to investigate associations between fatty acid fractions in breast adipose tissue and breast cancers [16–18]. Freed et al. showed a possible link between the invasive ductal carcinoma and fatty acid fractions in breast adipose tissue for postmenopausal women [19].

Here, we used ^1^H-MRS to study lipid composition and distribution in C3(1) SV40 large T-antigen (Tag) transgenic mice [20]. This is a well-established model of human triple-negative breast cancer, and has been used by other investigators to characterize the influence of diet on mammary fat and mammary cancer [21, 22]. This mouse model targets the expression of large T-antigen to the female mammary gland via the C3(1) promoter. These mice develop mammary cancer that resembles human breast ductal carcinoma, including progression through atypical ductal hyperplasia (~8 weeks), ductal carcinoma in situ (DCIS) (~12 weeks), and invasive ductal carcinoma (IDC) (~16 weeks) [23, 24].

The goal of the present study was to evaluate the impact of varying dietary composition on mammary fat in SV40 Tag mice. SV40 mice were fed either a low fat diet (LFD), high animal fat diet (HAFD), or high fructose diet (HFruD) and then evaluated 12 weeks of age using magnetic resonance imaging (MRI). This mouse strain and the time point for analysis were chosen so that the impact of diet on mammary fat could be studied without the confounding variables of generalized obesity and systemic metabolic disruptions [25]. Localized ^1^H-MRS was acquired at selected voxels in inguinal mammary gland tissue with no cancers, lymph nodes, or lymph ducts. In addition, high spectral and special resolution (HiSS) MRI data were acquired from nine slices to generate water and fat images. We hypothesize that the high fat diet, mainly due to lard, will create less PUFA in the SV40 mouse mammary gland at 12 weeks, before the age at which most of the invasive cancers appear.

## Materials and methods

### Animals

Female C3(1) SV40 Tag transgenic mice (n = 12), produced in Dr. Conzen’s laboratory, were studied and randomly divided into LFD (n = 4), HAFD (n = 4), and HFruD (n = 4) groups. The diets were started when mice were 4 weeks old and the mice were imaged at 12 weeks of age after 8 weeks on each diet. Food intake and individual mouse body weight were monitored weekly. The average (± standard deviation) body weight on the day of MRI experiments was 18.7 ± 0.9, 20.6 ± 1.9, and 20.1 ± 0.4 gram for LFD (3.8 kcal/g), HAFD (5.3 kcal/g), and HFruD (3.9 kcal/g) mice, respectively. Although the mice fed with HAFD and HFruD were not obese, they gained slightly more weight than the mice fed with LFD. These diets were purchased from Harlan Lab (Madison, WI, USA); Table 1 provides a list of ingredients in these three diets. Lard was treated with anti-oxidants to prevent spoilage; it is considered suitable for human consumption. The lard was also considered highly palatable to the mice as evidenced by the amount of food consumption and some weight gain in the animals fed the high fat diet.

**Table 1:** The detailed list of ingredients in low fat, high animal fat, and high fructose diets.

	Diet Components (g/Kg)		
Formula	Low fat	High animal fat	High fructose
Casein	200	200	200
L-Cystine	3	3	3
**Corn Starch**	397.5	117.4	--
Maltodextrin*	132	132	--
Sucrose	100	100	100
**Fructose**	--	--	529.4
Soybean oil	70	70	70
Lard**	--	280	--
Cellulose	50	50	50
Mineral Mix (AIN-93G-MX; 94046)	35	35	35
Vitamin Mix (AIN-93VX; 94047)	10	10	10
Choline Bitartrate	2.5	2.5	2.5
TBHQ (antioxidant)	0.014	0.014	0.014
Food Coloring***	--	0.1	0.1

* Due to the amount of fructose, both corn starch and maltodextrin had to be omitted from the diet to allow for inclusion of the fructose and supplementation with other dietary components.

** The maker of the diets was not able to provide details regarding the feed provided to the pigs that were the source of the lard, but they were most likely grain fed.

*** The food coloring is included to differentiate the experimental diets from the low fat diets during feeding.

All procedures were carried out with approval from the University of Chicago’s Institutional Animal Care and Use Committee. Animals were anesthetized prior to imaging experiments, and anesthesia was maintained during imaging between 1.5–2.5% isoflurane. Temperature, heart rate and respiration rate were monitored with a fiber optic detection system from SA Instruments (Stony Brook, NY, USA), designed for use in small animal MRI. The temperature was kept within normal range by using a temperature controlled fiber optic probe and heating system. The respiration rate was ~55 breaths per minute and used to obtain gated images.

### Imaging protocols

MRI experiments were performed on a 9.4 Tesla Bruker (Billerica, MA, USA) small animal scanner with 11.6 cm inner diameter, actively shielded gradient coils (maximum constant gradient strength for all axes: 230 mT/m). After being fitted with physiological monitoring equipment, mice were placed into a plastic semi-circular cradle and placed in a 30 mm diameter quadrature coil (Rapid MR International, Columbus, OH, USA). Axial high resolution multi-slice RARE (Rapid Acquisition with Relaxation Enhancement) spin echo T2-weighted (T2W) images (TR (repetition time)/TE (echo time)_effective_ = 4000/20.3 ms, FOV (field of view) = 25.6×19.2 mm, matrix size = 256×192, slice thickness = 0.5 mm, slice gap = 1 mm, number of slices = 31, NEX (number of excitations) = 2, RARE factor = 4) with fat suppression and respiratory gating were acquired over the mouse inguinal mammary gland. Two interleaved sets of images were acquired to cover the slice gaps and then combined for a total of 62 slices.

Based on T2W images, two 1 mm^3^ regions-of-interest (ROIs) were selected on each side of the inguinal mammary gland where there was no appearance of cancers, lymph nodes, or lymph ducts for ^1^H-MRS. Shimming was performed by iterative adjustment of global linear shims based on non-localized free induction decay (FID) integral. The localized ^1^H-MRS were acquired with a Bruker point resolved spectroscopy (PRESS) pulse sequence with no water suppression (TR/TE = 4000/11.8 ms, number of data points = 1024, spectral width = 6009.6 Hz, spectrum resolution = 2.9 Hz, NEX = 64) to follow the FID for 170.4 ms.

Finally, based on T2W scans, nine slices were selected for subsequent HiSS imaging to investigate more mammary gland area, because it was hard for localized ^1^H-MRS to cover the area. HiSS data were acquired using multiple gradient echoes with in-plane spatial resolution the same as T2W imaging (TR = 203 ms, first TE = 4.4 ms, subsequent echo spacing = 0.5 ms, number of echoes = 24, slice thickness = 1 mm, slice gap = 1 mm, nominal spectral resolution = 62.9 Hz, flip angle = 20°, NEX = 1) without fat suppression and respiratory gating.

### Data analysis

The MRI data were processed with software developed in MATLAB (MathWorks, Natick, Massachusetts, USA). For the ^1^H-MRS data, the free induction decay signals were Fourier-transformed, and the phase and the baseline of the spectra were corrected using TOPSPIN routine (Bruker, Billerica, MA, USA). Phasing was performed carefully to avoid errors due to mismatches between absorption and dispersion components. There was no line broadening applied to the spectral data.

Nine different lipid resonances were detected that corresponded to the ^1^H peaks in triacylglycerols. In order to accurately calculate the area under each resonance, the spectrum was fitted with a combination of Gaussian and Lorentz functions as follows [14, 26]:

$$s(f)=\sum_{n=1}^{10}\left[\alpha_{n}G_{n}(f)+(1-\alpha_{n})L_{n}(f)\right],$$ (1)

where f is frequency, α_n_ is constant, G_n_(f) is Gaussian function, and L_n_(f) is Lorentz function (n = 1 to 10 for nine fatty acid peaks and one water peak). The fits were optimized using the non-linear Levenberg-Marquardt algorithm. The area under each fat resonance was calculated based on fitted spectra.

To estimate mammary fat composition from ^1^H-MRS, peak area (PA) ratios were calculated as follows to obtain the poly-unsaturated fatty acid fraction (PUFA), mono-unsaturated fraction (MUFA), and saturated fatty acid fraction (SFA) based on a previous study [26]:

1. $$\text{PUFA}={\text{PA}}_{\text{2}\text{.8ppm}}/{\text{PA}}_{\text{2}\text{.3ppm}}$$ (2)
2. $$\text{MUFA}=\text{0}\text{.5}\cdot {\text{PA}}_{\text{2}\text{.1ppm}}/{\text{PA}}_{\text{2}\text{.3ppm}}-\text{PUFA}$$ (3)
3. SFA=1−(MUFA+PUFA) (4)

In addition, the mammary lipid quantity (MLQ) index [11] was determined as the ratio between the peak amplitude of the methylene (−(C**H**_2_)_n_−) peak at 1.3 ppm and the water peak at 4.7 ppm, i.e.,

$$\text{MLQ}={\text{PA}}_{\text{1}\text{.3ppm}}/\left({\text{PA}}_{\text{1}\text{.3ppm}}+{\text{PA}}_{\text{4}\text{.7ppm}}\right)$$ (5)

For the HiSS data sets, after the fat and water peaks were identified in all slices, the fat and water images (maps) were constructed from peak height of fat and water spectra, respectively. In order to compare results of different experiments, the water and fat peak height images (maps) were normalized by the average water peak height intensity over an ROI in back muscle. In order to evaluate fat content, the mammary gland ROI was manually traced on T2W images and superimposed on corresponding fat maps. Then, the average fat signal intensity over all mammary ROIs was calculated for each mouse. Only the pixels in the mammary gland with normalized signal intensity greater than 0.15 were considered to contain a significant fat signal, and were used in the calculation. The reason to use 0.15 instead of greater than 0.0 as threshold was to guarantee the pixels were fat.

The non-parametric Kruskal-Wallis (K-W) tests were performed to determine whether there was a significant difference in calculated parameters between mice on different diets. Then, pairwise Wilcoxon rank-sum tests were performed on the sample that was found to be statistically significant from the K-W tests. A p-value less than 0.05/3 (= 0.017), with a Bonferroni adjustment for multiple testing was considered significant.

## Results

**Fig 1:**
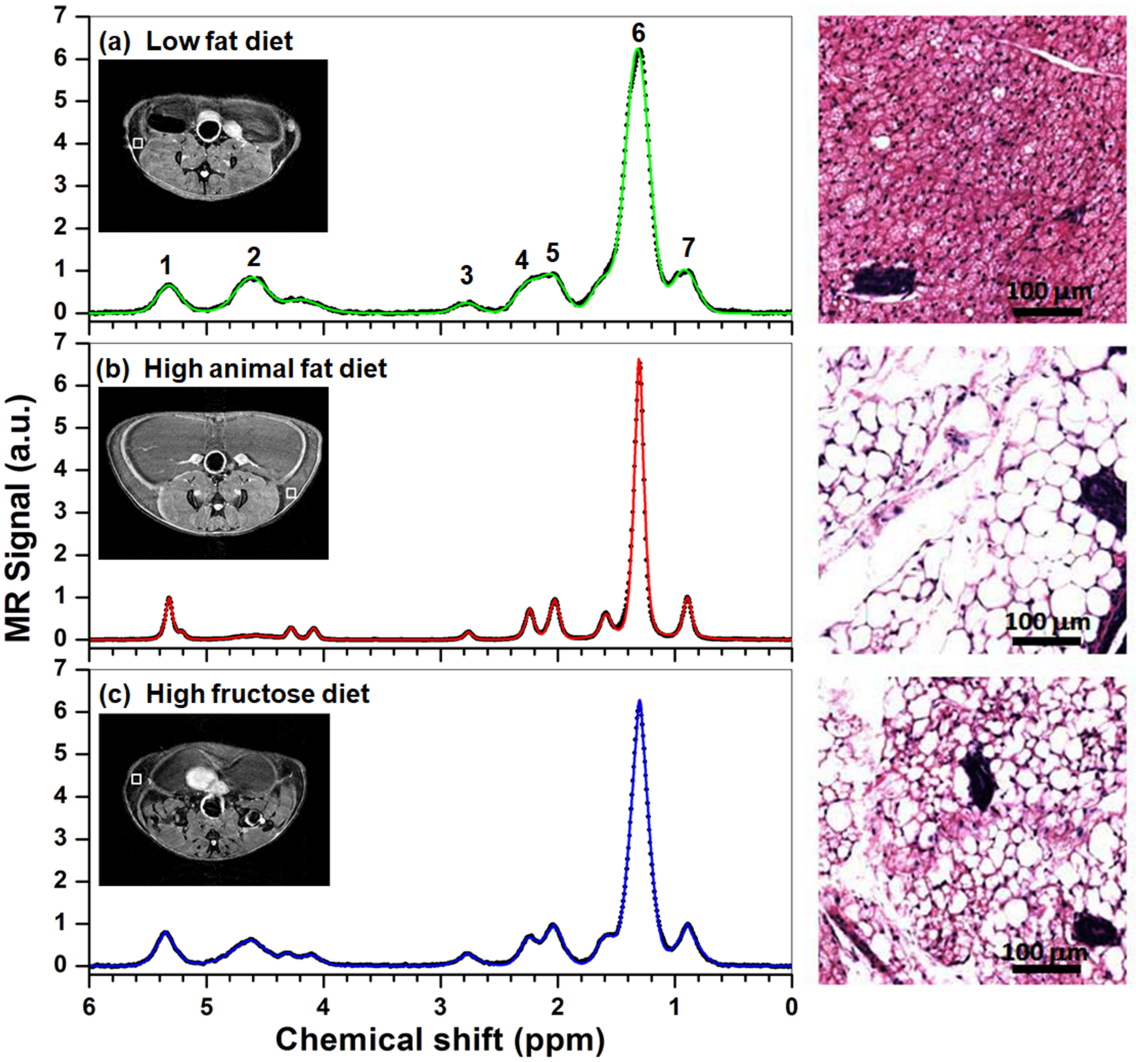
Plots of spectrum (black dots) measured from the LFD (top), HAFD (middle), and HFruD (bottom) fed SV40 mice, and corresponding fitted the spectrum (color lines) with a combination of Gaussian and Lorentz functions. 1: Olefin (−C**H** = C**H**−) at 5.4 ppm, 2: Water (**H**_2_O) at 4.65 ppm, 3: Diallyl (= CH−C**H**_2_−CH =) at 2.8 ppm, 4: α-Methylene to carboxyl at 2.3 ppm, 5: Allyl (−C**H**_2_−CH = CH−) at 2.1 ppm, 6: Methylene (−C**H**_2_−) at 1.3 ppm, 7: Methyl (−C**H**_3_) at 0.9 ppm. The locations of 1 mm^3^ ROIs (white squares) used for the PRESS pulse sequence are shown in the insets T2W images. The right panel shows examples of histological images from inguinal mammary glands.

To investigate the impact of dietary composition on mammary gland lipid storage, female C3(1) SV40 Tag transgenic mice were fed LFD, HAFD or HFruD for 8 weeks prior to MRI. Fig 1 shows three typical spectra (black dots) from mouse mammary glands after feeding with LFD (top), HAFD (middle) and HFruD (bottom). In each case the fit to the spectrum is shown as a colored line—each spectrum was fitted with a combination of Gaussian and Lorentz functions as shown in Eq 1. The locations of 1 mm^3^ ROIs (white squares) used for the PRESS pulse sequence are shown in the insets T2W images. Nine fatty acid peaks and one water peak were detected in the mammary gland. The spectrum from the LFD mouse is clearly different from the spectra from the HAFD and HFruD mice. The average (± standard deviation) full width at half maximum (FWHM) of the methylene peak (at 1.3 ppm) in LFD mice (0.21 ± 0.038 ppm) was significantly broader (p < 0.01) than in HAFD mice (0.12 ± 0.027 ppm) and in HFruD mice (0.16 ± 0.030 ppm). The increased linewidth in LFD mice compared to HAFD and HFruD mice cannot be attributed to systematic differences in shimming, since the average water resonance linewidth in lymph nodes near the selected PRESS boxes was narrower in LFD mice than in the other two groups. The average FWHM of the water proton magnitude spectrum measured from lymph nodes by HiSS was 0.25 ± 0.03 ppm for LFD mice, 0.30 ± 0.07 ppm for HAFD mice, and 0.27 ± 0.04 ppm for HFruD mice (these differences were not significantly different).

The increased fatty acid resonance linewidths in the LFD mice were consistent with histology of mouse mammary glands, showing that brown adipose tissue (BAT; known to increase proton linewidths [27]) predominated in LFD mice and mature white adipose tissue (WAT) was highly abundant in HAFD mice. Representative H&E-stained images of excised inguinal mammary glands in Fig 1 (right panel) show highly visible BAT containing numerous mitochondria in an LFD mouse, but the HAFD mammary gland contained primarily mature white fat distinguished by a single lipid droplet in the adipocyte. The brown fat content in HFruD mice was greater than in HAFD mice, but less than in LFD mice, as evident from H&E images in Fig 1. This pattern was seen on histology in all LFD, HAFD, and HFruD mice studied.

**Fig 2:**
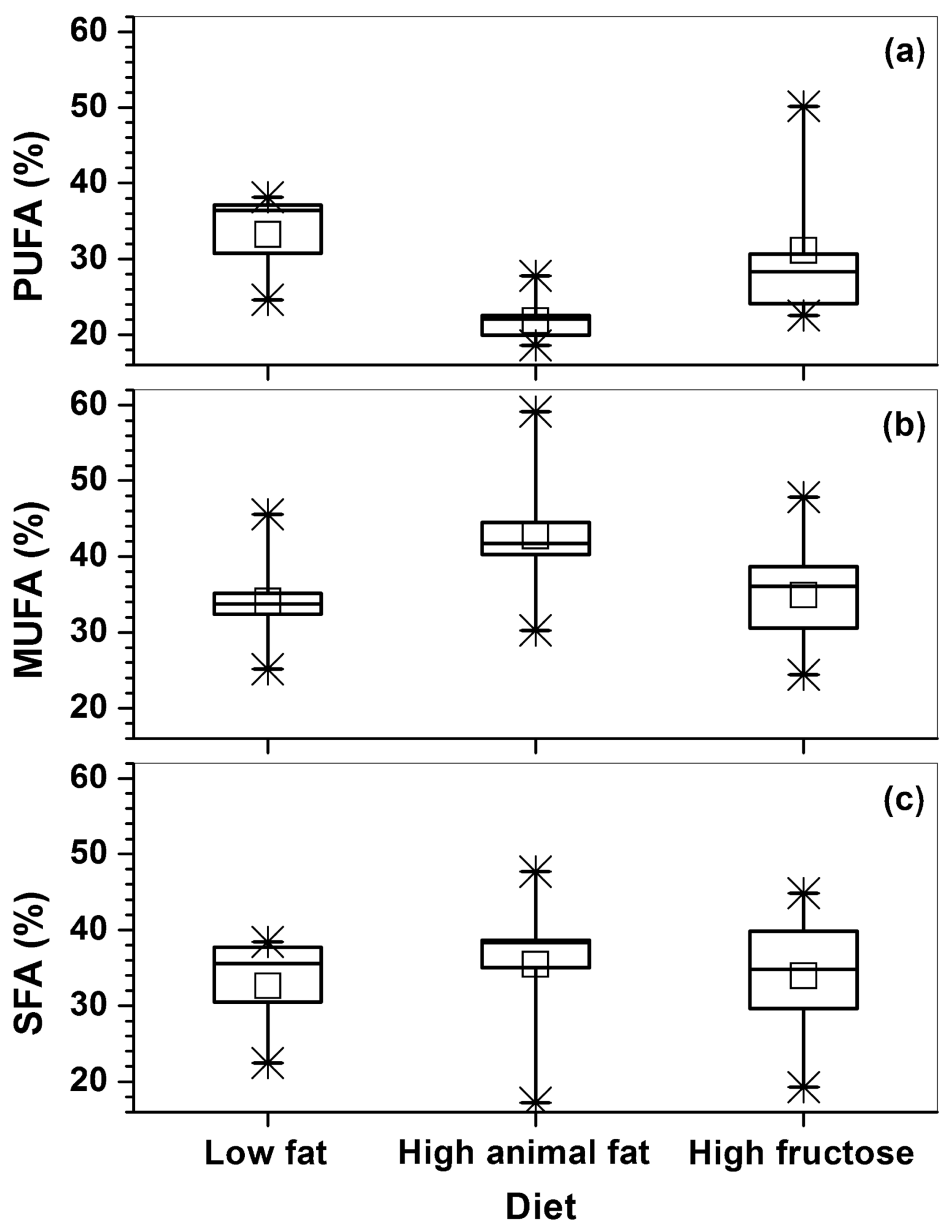
The box-plots of poly-unsaturated fatty acids (PUFA), mono-unsaturated fatty acids (MUFA), and saturated fatty acids (SFA) calculated from spectra acquired from the LFD, HAFD, and HFruD fed SV40 mice inguinal mammary glands. The squares (□) indicate mean, and the asterisks (*) indicate the upper and lower limits of the data.

**Fig 3:**
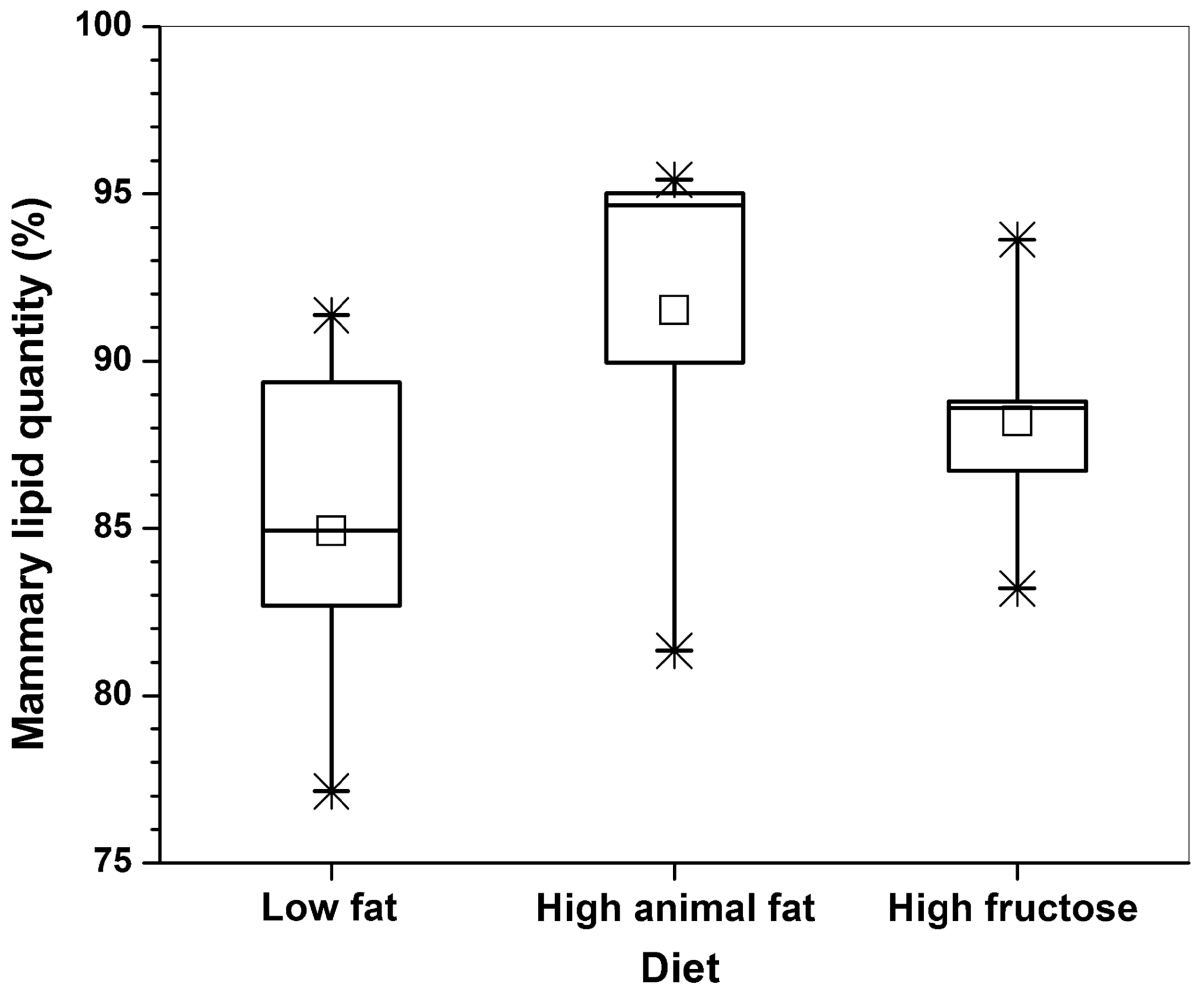
The box-plots of mammary lipid quantity calculated from spectra acquired from the LFD, HAFD, and HFruD fed SV40 mice inguinal mammary glands. The square (□) indicate mean, and the asterisks (*) indicate the upper and lower limits of the data.

Fig 2 shows the box-plots of calculated fatty acid contents of PUFA (Eq 2), MUFA (Eq 3), and SFA (Eq 4) based on 24 spectra measured from the three groups of diet mice. The statistical analysis showed that HAFD mice had a significantly lower poly-UFA than both LFD (p < 0.001) and HFruD (p < 0.01) mice. In addition, the HAFD mice had higher mono-UFA than both LFD (p = 0.04) and HFruD (p = 0.05) mice. However, there was no statistical difference (p = 0.1) in saturated-FA between the three groups of mice. Mammary glands of HAFD mice tended to contain greater ‘mammary lipid quantity’ (Fig 3) compared to both LFD (p = 0.03) and HFruD (p = 0.1) mice, but this difference was not statistically significant.

**Fig 4:**
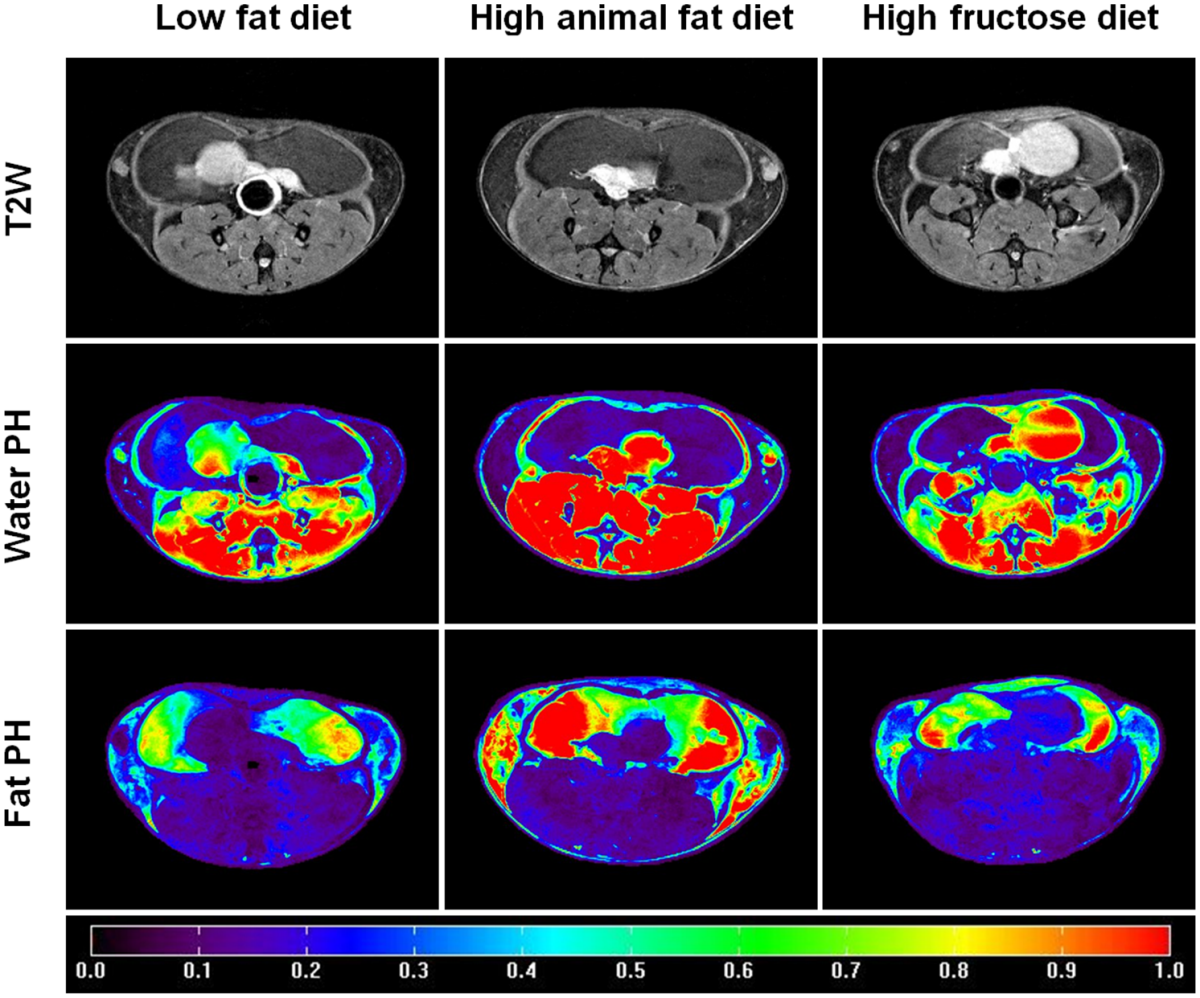
Example of T2W images (top row), water peak height (PH) images (maps) (middle row), and fat peak height (PH) images (maps) (bottom row) for LFD (left panel), HAFD (middle panel), and HFruD (right panel) fed SV40 mice. The color bar indicates the normalized signal intensity values, which were obtained by dividing the average of water peak height intensity over the muscle ROI.

Fig 4 shows an example of T2W images, water peak height images, and fat peak height images obtained from the HiSS data sets for three groups of mice. The average normalized fat signal intensity (mean ± standard deviation) over the mammary glands was 0.65 ± 0.11 for HAFD mice, which was higher than for LFD mice 0.40 ± 0.07 (p = 0.04) and HFruD mice 0.51 ± 0.05 (p = 0.08).

For all calculated parameters, the average (or median) values for HFruD mice fell between those for LFD and HAFD mice. For each calculated parameter, there was no statistical difference between HFruD and LFD mice or between HFruD and HAFD mice.

## Discussion

The impacts of different diets on mammary fat in a transgenic mouse model of human triple negative breast cancer were investigated using localized ^1^H-MRS and HiSS MRI. The results demonstrated that fat content increases consistently and fat composition changes significantly in the HAFD mice compared to both LFD and HFruD mice. Poly-UFA (“good fat”) was significantly lower in HAFD mice compared to both LFD and HFruD mice. Previous studies have highlighted the importance of PUFA assessment for breast cancer prevention and suggested that the different types of PUFAs, e.g., omega-6 and omega-3 have different roles in breast cancer proliferation [28, 29]. The omega-3 poly-UFA’s inhibit proliferation of breast cancer cells, while the omega-6 poly-UFA’s promote proliferation [28].

The average values of the PUFA (21.8%), MUFA (42.7%), and SFA (35.4%) fractions measured from the HAFD mice in this study were in the same range as those found in previous studies of human breast adipose tissue in patients with cancer on 3T and 7T (PUFA = 20.8–22.7%, MUFA = 48.5–55.4%, SFA = 23.8–28.7%) [16–18]. This suggested that the PUFA assessment could be used to guide the development and use of therapies that reduce mammary/breast cancer incidence. Using multi-gradient echo MRI, Freed et al. showed that the MUFA was significantly lower (38% vs. 46%) in postmenopausal women with invasive ductal carcinoma than in women with no known cancer [19].

Both localized ^1^H-MRS and HiSS MRI show that fat content increased and water content decreased in the HAFD mice compared to the LFD mice. Furthermore, results from histology show that brown adipose tissue (BAT) predominates in LFD mice, while mature white adipose tissue (WAT) is abundant in HAFD mice. This is illustrated in H&E images in Fig 1. BAT, in contrast to WAT, contains smaller adipocytes with multiple fat droplets, a centrally located nucleus, and an abundance of iron-rich mitochondria and intracellular water [30]. Because of that, BAT has higher water content and is associated with a reduction of T2*. The reduced T2* is consistent with the increased spectral linewidth (= 1/(T2*∙π)) found in mice on LFD as seen in the Fig 1. The FWHM of the methylene peak (the peak at 1.3 ppm) for HAFD mice was ~40% narrower than for LFD mice. This difference cannot be attributed to differences in shimming between the two groups of mice—since the average FWHM of the magnitude water resonance measured from lymph nodes by HiSS was narrower in LFD compared to HAFD mice (although this difference was not statistically significant). This is the opposite of the trend for fat resonances.

The results obtained for the high fructose diet were consistent with those of a previous study showing that consuming fructose-sweetened beverages increased body adiposity in mice [31]. A study of healthy human volunteers also demonstrated that four weeks of high fructose diet alters lipid metabolism [32]. Our results demonstrated a similar trend for the HFruD mice. The HiSS data analysis covering a much larger volume of mammary glands showed that fat content increased ~25% in HFruD mice compared to LFD mice, although this difference is not statistically significant, possibly due to small number of samples. The HFruD after weaning and during puberty may not only change fat content in subcutaneous and omental fat but also in the mammary gland. This is despite the diet fact that these mice were not obese.

There are several limitations to this study. First, the number of mice in each diet group was small; the results of this pilot study should be validated with a larger number of mice. Second, the number of PRESS spectra acquired over the mammary gland was small; future studies should include denser sampling of the mammary glands at high spectral resolution. Third, the spectral resolution and spectral bandwidth of HiSS data sets was modest; in future studies HiSS sampling methods will be improved to acquire data at higher spectral resolution with an acceptable run time, and to sample early echoes with shorter readout gradients in order to improve detection of short T2* signals [33]. Finally, these high fat and high fructose diets did not perfectly model Western diets. The nature and preservation of the lard could have impact on our observations.

## Conclusions

In summary, this study demonstrated that localized ^1^H-MRS and HiSS detect significant changes in mammary glands due to HAFD and HFruD. The lower poly-UFA may be an indication of cancer risk. Serial studies using these MRI methods could improve the understanding of the effects of diet on cancer incidence and progression. In addition, similar methods could be used in women to assess cancer risk and to guide preventative therapy, as suggested by the recent work of Freed et al. [19].
